# Large Enhancement of the Luminescence Properties of an Eu(III) Dye upon Association with the DNA-CTMA Matrix

**DOI:** 10.3390/molecules30061395

**Published:** 2025-03-20

**Authors:** Daniele Marinotto, Cosmina Andreea Marin, Ileana Rau, Alessia Colombo, Francesco Fagnani, Dominique Roberto, Claudia Dragonetti

**Affiliations:** 1 Istituto di Scienze e Tecnologie Chimiche (SCITEC) “Giulio Natta”, Consiglio Nazionale delle Ricerche (CNR), Via C. Golgi 19, 20133 Milan, Italy; 2 Faculty of Chemical Engineering and Biotechnologies, National University of Science and Technology POLITEHNICA Bucharest, 1-7 Polizu Street, 011061 Bucharest, Romania; lazarcosmina@ymail.com (C.A.M.);; 3 Secondary School “Nicolae Bălcescu”, Aleea Școlii 2, 100498 Ploieşti, Romania; 4 Secondary School “Nicolae Titulescu”, Popa Farcaș 23, 100058 Ploieşti, Romania; 5 Dipartimento di Chimica, Università degli Studi di Milano, UdR-INSTM, Via C. Golgi 19, 20133 Milan, Italydominique.roberto@unimi.it (D.R.);

**Keywords:** deoxyribonucleic acid (DNA), biopolymer, europium dye, sustainable materials, biodegradable matrices

## Abstract

In this study, the photophysical properties of thin films of an Eu^3+^ dye, namely europium tetrakis(dibenzoylmethide) triethylammonium (EuD_4_TEA), within deoxyribonucleic acid (DNA) biopolymer functionalized with hexadecyltrimethylammonium chloride (CTMA) were extensively investigated and compared with those of thin films of the same dye embedded in more conventional polymers, like poly(methyl methacrylate) and polycarbonate. The new materials obtained have good optical properties, as shown by their absorption and emission spectra. Remarkably, a large enhancement in photoluminescence was observed upon the interaction of EuD_4_TEA with DNA-CTMA (2- and 17-fold increase in luminescence quantum yield with respect to PMMA and PC). Photophysical analyses suggest that the emission enhancement was mainly due to the increase in the sensitization efficiency (η_sens_) from the ligands to the Eu^3+^ ion along with the suppression of the vibrational deactivation upon immobilization onto the DNA-CTMA matrix, as the concentration of the complex increased from 20 to 50%. These phenomena are primarily driven by the transformation of the Eu^3+^ micro-environments, which are created by the interactions between complex ligands and the DNA-CTMA matrix.

## 1. Introduction

In recent decades, eco-friendly biopolymers have attracted significant attention and spurred growing scientific research into their possible applications in different technological areas, owing to their versatility, biodegradability, and unique structural properties [1,2]. Among these biopolymers, deoxyribonucleic acid (DNA), which can be obtained from renewable resources, has proven to be particularly interesting as a low-cost material for photonic and optoelectronic devices since it exhibits characteristics that allow it to incorporate a variety of functional molecules such as organic dyes, coordination compounds, and conducting polymers through electrostatic binding, intercalation, and groove binding [3,4,5,6]. From a practical point of view, to ensure good processability, the thermal stability and photostability of thin film devices based on DNA biopolymer complexes between DNA and cationic surfactants have been realized [7,8]. By applying DNA–surfactant complexes, organic light emitting diodes (OLEDs) [9], photo-detectors [10], optical amplifiers [11,12,13,14], and organic transistors [15,16] have been reported.

Today, DNA-based materials are appealing as a sustainable technology, opening the doors to the fabrication of fascinating novel devices [17,18,19]. In particular, the solid matrices formed by the complexation of DNA with cetyltrimethylammonium chloride (CTMA) surfactant exhibit remarkable properties due to the synergistic effects arising from their interaction. Thus, the DNA-CTMA complex is characterized by high transmission in the near-infrared and visible regions of the spectrum, high charge carrier mobility, and excellent film-forming properties [8,20]. From an application point of view, an important aspect is that it is soluble in a large number of solvents, contrarily to pure DNA, which is soluble in water only, offering the possibility of functionalization with a large number of molecules which are water insoluble [21]. One of the most outstanding features of the DNA-CTMA complex is its ability to incorporate luminescent dyes with a high dopant concentration without having significant emission quenching, which makes it suitable for light-emitting devices [13]. Fluorescence quantum intensity is sometimes enhanced in the DNA-CTMA matrix, compared to the case in which the same dye is embedded in conventional organic polymers, like poly(methyl methacrylate) (PMMA), or in solution [21,22,23,24,25,26]. Remarkably, the DNA–CTMA matrix can largely suppress the concentration quenching of various metal complexes’ emissions due to the interactions between the luminescent compound and DNA-CTMA, which play a protective role in suppressing the vibrational and aggregational quenching of the luminescent compound [21,22,23,24,25,26].

**Scheme 1:**
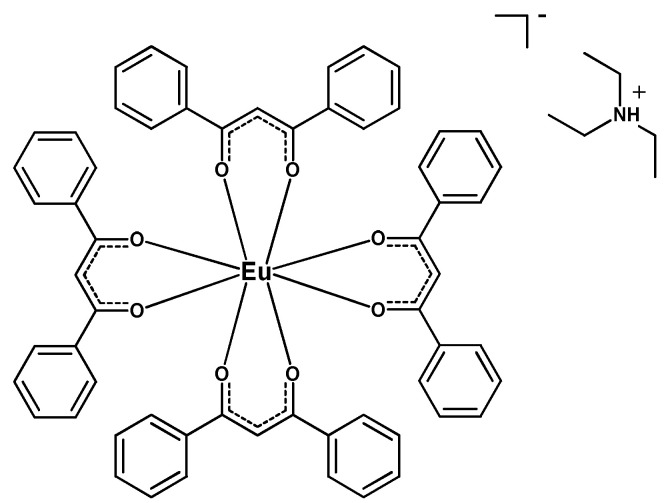
Structure of the europium tetrakis(dibenzoylmethide) triethylammonium dye.

Therefore, with the dual aim of (i) increasing the knowledge on the behavior of DNA–surfactant complexes as host matrices for dyes in photonic and optoelectronic devices and (ii) developing new DNA-based photo-functional materials, this paper focuses on the introduction of the luminescent europium tetrakis(dibenzoylmethide) triethylammonium (EuD_4_TEA, Scheme 1) dye [27,28,29] into a DNA-CTMA matrix.

The choice of EuD_4_TEA as a dye is novel with respect to previous works [21,22,23,24,25,26]. It has been chosen in view of its excellent optical properties as a luminophore, with a good absorption spectral breadth in the UV region, narrow emission bands with large Stokes shifts, and long phosphorescence lifetimes [29]. Moreover, due to the fine structure and the relative intensities of the transitions in the luminescence spectra, EuD_4_TEA dye can be used as a structural probe, deciphering the symmetry of the chemical environment and, partly, the composition of the inner coordination sphere of the Eu^3+^ center in the DNA-CTMA matrix [30,31].

Thus, red-luminescent composite thin films containing DNA-CTMA and EuD_4_TEA with various ratios were prepared and, for comparison, PMMA and polycarbonate (PC) polymers were also used as host materials. It is worth pointing out that a direct comparison of the influence of the DNA-CTMA matrix with that of PMMA is usually found in the literature [22,24,26], but not with PC. Detailed photophysical investigations of the influence of the interaction between DNA-CTMA, PMMA, and PC and EuD_4_TEA dye have been carried out.

## 2. Results and Discussion

**Figure 1:**
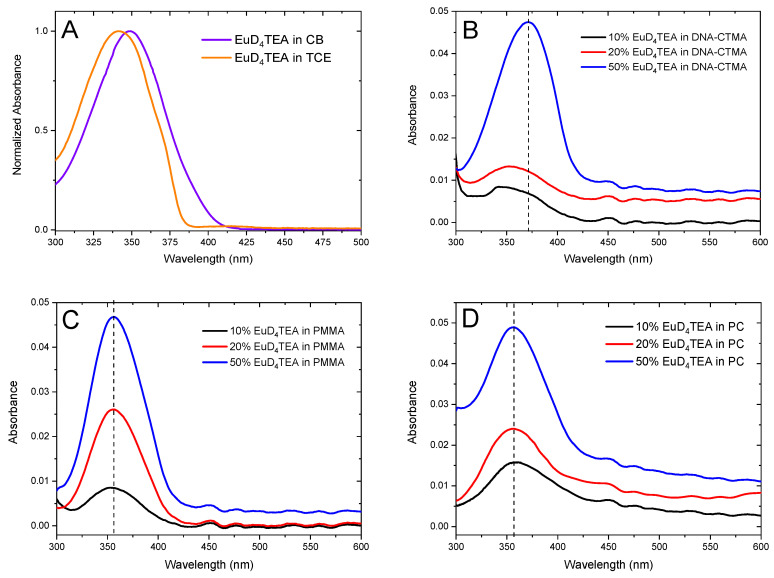
Panel (**A**): normalized absorption spectra of EuD_4_TEA in TCE and CB solutions. Panels (**B**–**D**): absorption spectra of thin films of EuD_4_TEA at various concentrations in DNA-CTMA, PMMA, and PC matrices, respectively. The dotted lines mark the absorption maxima of the samples.

Figure 1A shows the normalized absorption spectra of EuD_4_TEA dye in solution (chloroform–butanol 1:0.7 *v*/*v* -CB- and 1,1,2-trichloroethane-TCE-). Both spectra display a broad absorption band in the 300–425 nm spectral range, which is related to the π-π* electronic transition of the organic ligand dibenzoylmethide of the Eu complex [32,33], with a wavelength of maximum absorption peak (λ_abs,max_) at 349 and 343 nm for CB and TCE, respectively. Figure S1 compares the absorption spectra of EuD_4_TEA in solution at different concentrations, both with CB in the presence of DNA–CTMA and with TCE in the presence of PMMA or PC.

For TCE solutions containing PMMA or PC polymers, no significant variations in the positions of the absorption bands (λ_abs,max_ = 343 nm) are observed when the concentration of the complex is increased; moreover, the intensities of the absorption bands almost follow the Lambert–Beer law, indicating very weak interactions between chromophores and the polymeric matrices. However, in the case of the CB solvent with DNA-CTMA, a slight shift at 345 nm is noted in the position of the absorption bands of the more concentrated solutions; furthermore, increasing the concentration of the complex the Lambert–Beer law is no longer respected. No aggregates are observed in the absorption spectra. These behaviors suggest a strong interaction between chromophores and the polymeric matrix, possibly due to the intercalation or semi-intercalation of the complex in the DNA backbone [5].

The absorption spectra of the composite thin films of EuD_4_TEA at various loadings in DNA-CTMA, PMMA, and PC polymers are shown in Figure 1B, Figure 1C, and Figure 1D, respectively. As already observed in solution, when increasing the loading of the Eu dye, the films in PMMA and PC matrix do not display variations in the position of the absorption bands (λ_abs,max_ = 356 nm). On the other hand, the absorption peak of the DNA-CTMA/EuD_4_TEA film is red-shifted at 371 nm by about 15 nm, providing evidence of a strong interaction between the DNA-CTMA matrix and the EuD_4_TEA complex, which could reasonably be due to a possible intercalation of EuD_4_TEA in the DNA helix, by analogy with other metal complexes [5,26,34,35]. Thus, an intercalative binding mode concerning π-stacking interaction between the dibenzoylmethide ligand and DNA base pairs could occur, as previously reported for dibenzoylmethide silver compounds and dibenzoyl methane itself [36].

In order to discuss the influence of the interaction between EuD_4_TEA and the DNA-CTMA matrix on the luminescent properties and emission and excitation spectra, lifetime and quantum yield measurements were carried out on DNA-CTMA/EuD_4_TEA films and, for comparison, on PMMA/EuD_4_TEA and PC/EuD_4_TEA thin films also.

**Figure 2:**
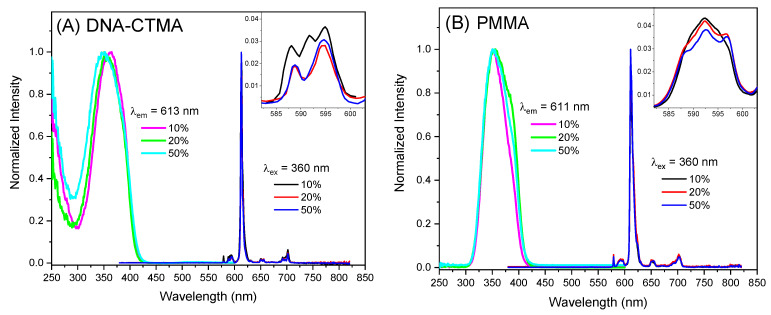

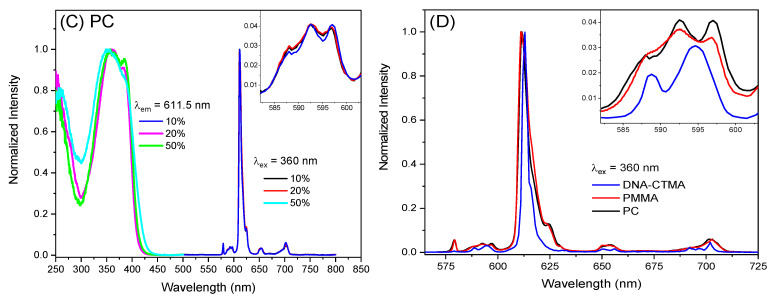
Panels (**A**–**C**): normalized excitation and emission spectra of thin films of EuD_4_TEA at various concentrations in DNA-CTMA, PMMA, and PC matrices, respectively. Panel (**D**): comparison of the 50% *w*/*w* films in the three different matrices. In each inset, the magnification of the spectrum refers to the ^5^D_0_ → ^7^F_1_ transition.

The normalized emission and excitation spectra of the thin films of the DNA-CTMA, PMMA, and PC matrices incorporating EuD_4_TEA with different concentrations at 298 K are shown in Figure 2. All films display a red emission with sharp peaks related to the f–f transitions of the Eu^3+^ ion upon ligand excitation (360 nm). In particular, five emission bands can be seen in each spectrum at about 578, 590, 613, 651, and 698 nm, which are attributed to the ^5^D_0_ → ^7^F_0,1,2,3,4_ transitions, respectively; the remaining transitions ^5^D_0_ → ^7^F_5_ and ^5^D_0_ → ^7^F_6_ at higher wavelengths are too weak to be observed. Moreover, no phosphorescence of the ligand is detected in the emission spectra.

In all emission spectra, the intensity of the so-called ”hypersensitive transition” (^5^D_0_ → ^7^F_2_) is greater than that of the other peaks in the spectrum, and it does not change position as the loading of the complex in the three matrices is increased (Figure 2, Panels A–C). In addition, the peak of the ^5^D_0_ → ^7^F_2_ transition is blue-shifted at 613.0, 611.5 nm, and 611.0 nm as EuD_4_TEA is embedded in DNA-CTMA, PC, and PMMA matrices, respectively (Figure 2, Panel D).

The excitation spectra of EuD_4_TEA in the three matrices were acquired at the maximum wavelength of the emission spectrum; see Figure 2. They are essentially identical in their respective absorption spectra; therefore, it is reasonable to assume that the organic ligand acts as a sensitizer in which the complex undergoes an intramolecular energy transfer from the excited triplet state of the ligand to an excited level of the Eu^3+^ ion.

The ^5^D_0_ → ^7^F_1_ band is a magnetic dipole (MD) transition whose intensity is mostly independent of the environment of the Eu^3+^ ion, and it can be used as an “internal reference” to compare the intensity of different emission spectra (see Figure S2). Once the integrated total intensity of the ^5^D_0_ → ^7^F_1_ band had been used to calibrate the intensity of the emission spectra, we took the integrated intensity ratio (R) of the transitions ^5^D_0_ → ^7^F_2_ and ^5^D_0_ → ^7^F_1_, R = I(^5^D_0_ → ^7^F_2_)/I(^5^D_0_ → ^7^F_1_) as a measure of the asymmetry of the first coordination sphere of the Eu^3+^ site in the three matrices (see Table 1). Specifically, the asymmetry of the Eu^3+^ site depends on the nature of the ligands and their interaction with the matrix. For the PC matrix, R ranges around 15.9, 15.7, and 17.6, increasing slightly with the loading of the complex. However, for the PMMA and DNA-CTMA matrices, the increase in R is higher, at 16.9, 17.9, 20.2 and 15.0, 20.5, 19.8, respectively. According to a study by Minami et al., in which the europium tris[3-(trifluoromethylhydroxymethylene)-(+)-camphorate] complex was embedded in PMMA and DNA-CTMA matrices, the resulting R value was much smaller, at 7.64 and 5.60, respectively [22], suggesting that the environments of the Eu^3+^ ion in the present work are the result of much stronger interactions between the complex ligands and the matrices.

It is noteworthy that for films with the DNA-CTMA matrix, an increase in the complex loading from 10% to 20% causes a very strong change in R and therefore in the Eu^3+^ micro-environment, after which R tends to stabilize. By contrast, for films based on PMMA and PC matrices, R increases gradually and no sudden jumps are observed. These behaviors are also confirmed by the crystal-field splitting of the ^7^F_1_ level, in which the ^5^D_0_ → ^7^F_1_ band in the DNA-CTMA film at 10% displays a greater number of visible components with respect to 20 and 50%; meanwhile for PMMA and PC, three components are always shown (see the inset in Figure 2, Panels A–C). It is known that the lower the symmetry class of the first coordination sphere of the Eu^3+^ site, the greater the number of visible components of the ^5^D_0_ → ^7^F_1_ transition [30]. Therefore, by changing the loading of the complex in the DNA-CTMA matrix, the Eu^3+^ micro-environments experience a different symmetry class, which is a direct consequence of the different interactions of the ligands with the matrix molecules. This is a further confirmation of the strong interaction between the Eu dye and the DNA-CTMA matrix, such as a possible intercalation of the EuD_4_TEA complex in the DNA helix due to π-stacking interaction between the dibenzoylmethide ligand and DNA base pairs [36], as previously hypothesized in the analysis of the absorption spectra following the increase in the dye loading. By contrast, for the PMMA and PC matrices, the interactions of the ligands with the matrix molecules do not lead to a change in the symmetry class.

Time-resolved fluorescence decays of EuD_4_TEA embedded in the DNA-CTMA, PMMA, and PC matrices measured at the wavelength of the maximum of the emission spectrum, exciting at 360 nm, are shown in Figures S3–S11, and the average lifetimes (*τ_av_*) are summarized in Table 1. The luminescent decay curves of all films are fitted by bi-exponential functions; see ESI for more details. Although two kinds of luminescent lifetimes can be associated with the emission from the ^5^D_1_ and ^5^D_0_ levels resulting from the superposition between the ^5^D_1_ → ^7^F_4_ and ^5^D_0_ → ^7^F_2_ transitions [30,37], this possibility can be excluded because emission bands from a higher excited state are not observed in the emission spectra. Therefore, the two lifetimes could be reasonably attributed to the heterogeneities of the Eu^3+^ micro-environments, which are created by the interactions between the complex ligands and the matrices.

From the data in Table 1, the average lifetime decreases with increasing concentrations of EuD_4_TEA dye in the films of the PMMA and PC matrices, while for the films in the DNA-CTMA matrix, the *τ_av_* decreases from 10 to 20% and then increases slightly when the loading reaches 50%. Looking at the two “populations”, i.e., the amplitudes of the two pre-exponential coefficients (α_1_ and α_2_) of the fitting exponential function in Table S1, it is possible to recognize how there is a sudden and strong increase in the α_2_ population of the longer lifetime from 10 to 20% at the expense of the shorter one (α_1_), at just the same dye loading in which the Eu^3+^ sites experience a change in symmetry due to the strong interaction of EuD_4_TEA in the DNA-CTMA matrix.

In order to establish the luminescence efficiency of the EuD_4_TEA dye embedded in the DNA-CTMA, PMMA, and PC matrices, the overall quantum yield (*Φ_tot_*) was determined after excitation into the absorbing bands of the ligands at 360 nm (see Table 1). The *Φ_tot_* increases as the loading of the EuD_4_TEA dye in the three matrices increases, and it is within 17.3–25.1, 9.2–12.2, and 1.1–1.5% for the films in DNA-CTMA, PMMA, and PC, respectively. Interestingly, the *Φ_tot_* is much higher in the DNA-CTMA than in the PMMA and PC matrices; specifically, for films with 50% *w*/*w* loading, the increase occurs by a factor of approximately 2 and 17, respectively. This demonstrates how a large concentration of EuD_4_TEA dye can be included into the DNA-CTMA matrix without causing appreciable emission quenching.

To highlight the factors that contributed to the emission enhancement of EuD_4_TEA in the DNA-CTMA films, we calculated the radiative (*k_r_*) and non-radiative (*k_nr_*) rate constants, intrinsic quantum yield (*Φ_Ln_*), and ligand sensitization efficiency (*η_sens_*). In particular, the radiative rate constants were calculated using the following relation:

$$k_{r}=A_{MD,0}\;{\times n}^{3}\times \left(\frac{I_{tot}}{I_{MD}}\right)$$

where *A_MD_*_,0_ is the spontaneous emission coefficient of the ^5^D_0_ → ^7^F_1_ transition (= 14.65 s^−1^), *n* is the refractive index of the medium, and *I_tot_*/*I_MD_* is the ratio of the integrated radiation corresponding to the ^5^D_0_ → ^7^F_j_ transition (j = 0–6) to the peak area corresponding to the ^5^D_0_ → ^7^F_1_ transition [38,39,40,41]. If the transitions ^5^D_0_ → ^7^F_5_ and ^5^D_0_ → ^7^F_6_ are not included in the integration, an error of a few % will be made. Here, the value of *n* was determined to be 1.52, 1.49, and 1.575 for the DNA-CTMA [42], PMMA [43], and PC [44] films, respectively. In addition, the non-radiative rate constants (*k_nr_*) were calculated from the following relations:

$$\tau_{av}=\frac{1}{k_{r}+k_{nr}},\;{\;k}_{nr}=\frac{1}{\tau_{av}}-k_{r}$$

Meanwhile, the intrinsic quantum yield (*Φ_Ln_*) and efficiency of sensitization of the lanthanide luminescence by the ligand (*η_sens_*) were calculated from the following relations [38]:

$$\Phi_{Ln}=\frac{k_{r}}{k_{r}+k_{nr}},\;\eta_{sens}=\frac{\Phi_{tot}}{\Phi_{Ln}}$$

These photophysical parameters are listed in Table 1. It is evident that the probability of light emission from the excited states of EuD_4_TEA in the three matrices, i.e., the *k_r_* values, are very similar: 937, 1230, 1180 s^−1^ for DNA-CTMA; 984, 1041, 1159 s^−1^ for PMMA and 1111, 1106, 1223 s^−1^ for PC. Moreover, *k_r_* only slightly changes when varying the concentration of the dye. Differently, the non-radiative rate constants show lower values for PMMA (1066, 1384, 1564 s^−1^), and increase for DNA-CTMA (2208, 2831, 2542 s^−1^) and PC (1978, 2223, 3191 s^−1^). The increase in *k_nr_* clearly indicates a better vibronic coupling between the excited states of the Eu^3+^ ion and the molecules of the DNA-CTMA and PC matrices; however, it is worth pointing out that as the concentration of the complex in the DNA-CTMA matrix increases from 20 to 50%, and *k_nr_* decreases. Clearly, the detrimental molecular vibrations can be suppressed when the molecules of the dye interact more strongly with the DNA-CTMA matrix, as already observed upon the intercalation or semi-intercalation of other complexes in a DNA structure [22,45].

The intrinsic quantum yield (*Φ_Ln_*) represents the quantum yield that should be observed for direct excitation in the 4f levels of the Eu^3+^ ion, and by definition it is closely related to the *k_r_* and *k_nr_* values. *Φ_Ln_* in the films of the PMMA matrix displays higher values than in the films of the PC and DNA-CTMA matrices. However, the most important aspect is that as the loading increases, *Φ_Ln_* decreases for the films in the PMMA and PC matrices, whereas it increases for DNA-CTMA. In addition, the *η_sens_* value of EuD_4_TEA is significantly improved upon interaction with DNA-CTMA; it is almost 2.8 and 14.6 times higher compared to that of PMMA and PC, respectively. This improvement in *η_sens_* is believed to contribute to the enhancement of the emission of the EuD_4_TEA dye. As a final observation, it is important to underline that the strongest increase in increasing *η_sens_* from 58.1 to 78.2% is obtained when the loading in the film goes from 10 to 20%.

## 3. Materials and Methods

### 3.1. Materials Preparation

Reagents and solvents were provided by Sigma-Aldrich (St. Louis, MO, USA), unless otherwise evidenced, while complex EuD_4_TEA (europium tetrakis(dibenzoylmethide) triethylammonium) was synthesized as previously reported [29]: Eu(NO_3_)_3_·5H_2_O (330 mg, 0.77 mmol) and NEt_3_ (1 mL, 7.21 mmol) were added to a hot solution of dibenzoylmethane (700 mg, 3.125 mmol) in absolute EtOH (15 mL). The solution was heated for 1 h and then allowed to cool overnight. The crystals, formed upon cooling, were washed with EtOH (5 mL) and air dried, giving the product as a pale-yellow solid (758 mg; yield 85%).

Deoxyribonucleic acid (DNA), extracted from salmon processing waste, with 96% purity was purchased from Chitose Institute of Science & Technology, Chitose, Japan. DNA was functionalized with a cationic surfactant, hexadecyltrimethylammonium chloride (CTMA, 99% purity, Acros Organics, The Hague, The Netherlands), in order to solubilize it in organic solvent (*n*BuOH), as previously reported [21].

Thin films were obtained by spin-coating from solutions, obtained by dissolving the appropriate amounts (% *w*/*w*) of dry masses. Depending on the matrix and on the dye solubility, the solvents used were 1,1,2-trichloroethane (TCE, for PMMA and PC) or a mixture of chloroform and butanol (1:0.7 *v*/*v* chloroform–butanol, for DNA-CTMA).

### 3.2. Spectroscopic Measurements

UV-visible absorption spectra were recorded by an Evolution 220 UV-Visible spectrophotometer (Thermo Scientific™, Waltham, MA, USA). Steady-state emission and excitation spectra, and photoluminescence lifetimes, were obtained using an FLS 980 (Edinburg Instrument Ltd., Livingston, UK), in which emission spectra were corrected for the quantum efficiency of the photomultiplier tube and background intensity. Excitation spectra were adjusted for the intensity fluctuation in the Xenon lamp. Absolute photoluminescence quantum yields were measured using a C11347 Quantaurus (Hamamatsu Photonics K.K., Hamamatsu, Japan) [46].

## 4. Conclusions

For the first time, red-luminescent composite thin films containing EuD_4_TEA dye at various loadings in DNA-CTMA, PMMA and PC polymers were prepared, and detailed photophysical investigations were carried out. A much larger emission enhancement (*Φ_tot_*) was observed upon interaction with the DNA-CTMA matrix with respect to the PMMA matrix, as previously observed for other dyes [22,24,26], or to a PC matrix. Thus, with a 50% wt loading of EuD_4_TEA dye, the *Φ_tot_* of the film based on the DNA-CTMA matrix was enhanced by a factor of approximately 2- and 17-fold with respect to matrices based on PMMA and PC, respectively. Remarkably, a large concentration of EuD_4_TEA dye may be included in the DNA-CTMA matrix without causing appreciable emission quenching. The photophysical analyses suggest that the emission enhancement was mainly due to the increase in the sensitization efficiency (*η_sens_*) from the ligands to the Eu^3+^ ion, along with the suppression of vibrational deactivation upon immobilization onto the DNA-CTMA matrix as the concentration of the dye increases from 20 to 50%. These phenomena are primarily driven by the transformation of the Eu^3+^ micro-environments, which are created by the interactions between the complex ligands and the DNA-CTMA matrix. Further work is needed in order to obtain a better understanding of the nature of these interactions. In any case, it is reasonable to anticipate that such enhancements of the optical properties of EuD_4_TEA dye in a DNA-CTMA matrix can contribute to the creation and development of new luminescent devices based on DNA, with applications in biological fields and DNA engineering.

## Figures and Tables

**Table 1:** Photophysical data of films made with complex EuD_4_TEA at various concentrations in PC, PMMA, and DNA-CTMA matrices.

Sample	Eu–Polymer	R	*τ_av_*/μs	*k_r_*/s^−1^	*k_nr_*/s^−1^	*Φ_Ln_*/%	*η_sens_*/%	*Φ_tot_*/%
**PC/Eu**	1:2 (50% *w*/*w*)	17.6	226.55	1223	3191	27.7	5.4	1.5
	1:5 (20% *w*/*w*)	15.7	300.38	1106	2223	33.2	3.3	1.1
	1:10 (10% *w*/*w*)	15.9	323.74	1111	1978	35.9	3.1	1.1
**PMMA/Eu**	1:2 (50% *w*/*w*)	20.2	367.28	1159	1564	42.6	28.7	12.2
	1:5 (20% *w*/*w*)	17.9	412.52	1041	1384	42.9	26.1	11.2
	1:10 (10% *w*/*w*)	16.9	487.91	984	1066	48.0	19.2	9.2
**DNA-CTMA/Eu**	1:2 (50% *w*/*w*)	19.8	268.63	1180	2542	31.7	79.2	25.1
	1:5 (20% *w*/*w*)	20.5	246.20	1230	2831	30.3	78.2	23.7
	1:10 (10% *w*/*w*)	15.0	318.68	937	2208	29.8	58.1	17.3

## Data Availability

The data supporting this article have been included as part of the Supplementary Information.

## Supplementary Materials

The following supporting information can be downloaded at: https://www.mdpi.com/article/10.3390/molecules30061395/s1. Figure S1: Panel A, B and C: solutions of EuD_4_TEA complex at different concentrations in the TCE and CB solvents in presence of DNA–CTMA, PMMA, and PC polymers, respectively; Figure S2: Panel A, B, and C: emission spectra of thin films of the EuD_4_TEA complex at various concentrations in DNA-CTMA, PMMA, and PC matrices, respectively. For all three matrices, the spectra have been scaled such that the respective ^5^D_0_ → ^7^F_1_ bands have identical areas.; Figure S3: Time-resolved fluorescence decays and the relative fit of the 10% EuD_4_TEA complex in the DNA-CTMA matrix. Emission wavelength 613 nm; excitation wavelength 360 nm; Figure S4: Time-resolved fluorescence decays and the relative fit of the 20% EuD_4_TEA complex in the DNA-CTMA matrix. Emission wavelength 613 nm; excitation wavelength 360 nm; Figure S5: Time-resolved fluorescence decays and the relative fit of the 50% EuD_4_TEA complex in the DNA-CTMA matrix. Emission wavelength 613 nm; excitation wavelength 360 nm; Figure S6: Time-resolved fluorescence decays and the relative fit of the 10% EuD_4_TEA complex in the PMMA matrix. Emission wavelength 611 nm; excitation wavelength 360 nm; Figure S7: Time-resolved fluorescence decays and the relative fit of the 20% EuD_4_TEA complex in the PMMA matrix. Emission wavelength 611 nm; excitation wavelength 360 nm; Figure S8: Time-resolved fluorescence decays and the relative fit of the 50% EuD_4_TEA complex in the PMMA matrix. Emission wavelength 611 nm; excitation wavelength 360 nm; Figure S9: Time-resolved fluorescence decays and the relative fit of the 10% EuD4TEA complex in the PC matrix. Emission wavelength 611.5 nm; excitation wavelength 360 nm; Figure S10: Time-resolved fluorescence decays and the relative fit of the 20% EuD_4_TEA complex in the PC matrix. Emission wavelength 611.5 nm; excitation wavelength 360 nm; Figure S11: Time-resolved fluorescence decays and the relative fit of the 50% EuD_4_TEA complex in the PC matrix. Emission wavelength 611.5 nm; excitation wavelength 360 nm; Table S1: Lifetimes and amplitude of the two pre-exponential coefficients (α1 and α2) of the fitting exponential functions of the thin films of the EuD_4_TEA complex at various concentrations in DNA-CTMA, PMMA, and PC matrices.

## Abbreviations

The following abbreviations are used in this manuscript:

TCE	1,1,2-trichloroethane
CB	Chloroform–butanol (1:0.7 *v*/*v*)
EuD_4_TEA	europium tetrakis(dibenzoylmethide) triethylammonium
DNA	deoxyribonucleic acid
CTMA	hexadecyltrimethylammonium chloride
PMMA	poly(methyl methacrylate)
PC	polycarbonate
