# Supplementary material for: Large Enhancement of the Luminescence Properties of an Eu(III) Dye upon Association with the DNA-CTMA Matrix
Source: Molecules. 2025 Mar 20;30(6):1395. doi: 10.3390/molecules30061395 (PMC11944783; doi:10.3390/molecules30061395)
Supplement: Supplementary file 1 [file molecules-30-01395-s001.zip › molecules-3504156-supplementary.pdf]

# Large enhancement of the luminescence properties of an Eu(III) dye upon association with the DNA-CTMA matrix

Daniele Marinotto [1,*], Cosmina Andreea Marin [2,3], Ileana Rau [2], Alessia Colombo [4], Francesco Fagnani [4,*], Dominique Roberto [4] and Claudia Dragonetti [4]

[1] Istituto di Scienze e Tecnologie Chimiche (SCITEC) "Giulio Natta", Consiglio Nazionale delle Ricerche (CNR), via C. Golgi 19, 20133 Milan, Italy

[2] Faculty of Chemical Engineering and Biotechnologies, National University of Science and Technology POLITEHNICA Bucharest, 1-7 Polizu Street, 011061 Bucharest, Romania

[3] Secondary School "Nicolae Bălcescu", Aleea Școlii 2, 100498 Ploieşti, Romania; Secondary School "Nicolae Tit-ulescu", Popa Farcaș 23, 100058 Ploieşti, Romania

[4] Dipartimento di Chimica, Università degli Studi di Milano, UdR-INSTM, Via C. Golgi 19, 20133 Milan, Italy

* Correspondence: daniele.marinotto@cnr.scitec.it (D.M.), francesco.fagnani@unimi.it (F.F.)

# Contents


I. General Information S1

II. Photophysical data in solution S2

III. Photophysical data in thin films S3

IV. References S8


# I. General Information

Luminescence measurements were carried out on quartz substrate.

Absolute photoluminescence quantum yield (Φ) for thin film was measured using a C11347 Quantaurus Hamamatsu Photonics K.K spectrometer. A description of the experimental setup and measurement method can be found in the article of K. Suzuki et al [1]. Φ was calculated through Equation:

$$\Phi = \frac{PN\ (Em)}{PN\ (Abs)} = \frac{\int \frac{\lambda}{hc}\left[I_{em}^{sample}(\lambda) - I_{em}^{reference}(\lambda)\right]d\lambda}{\int \frac{\lambda}{hc}\left[I_{exc}^{sample}(\lambda) - I_{exc}^{reference}(\lambda)\right]d\lambda}$$

where PN(Em) is the number of emitted photons, PN(Abs) the number of absorbed photons, λ the wavelength, h the Planck's constant, c the speed of light, $I_{em}^{sample}$ and $I_{em}^{reference}$ the photoluminescence intensities of the sample and reference, $I_{exc}^{sample}$ and $I_{exc}^{reference}$ the excitation light intensities of the sample and reference. PN(Em) is calculated in the wavelength interval [$\lambda_i$, $\lambda_f$], where $\lambda_i$ is taken at about 10 nm above the excitation wavelength, while $\lambda_f$ is the upper end wavelength in the emission spectrum. The error made was estimated at around 5%.

Time-resolved fluorescence curves were fitted by a multi-exponential function:

$$I(t) = \sum_{i=1}^{m} \alpha_i \exp\left(\frac{-t}{\tau_i}\right)$$

where m is the number of exponentials, $\alpha_i$ is the pre-exponential factor and $\tau_i$ is the lifetime of the component i. The quality of the fit was evaluated through the reduced $\chi^2$ value.

In case of multi-exponential decay, it is possible to define an average lifetime as:

$\tau_{av} = \frac{\sum_{i=1}^{m} \alpha_i \tau_i^2}{\sum_{i=1}^{m} \alpha_i \tau_i}$ m = multi-exponential decay number of the fit.

## II. Photophysical data in solution

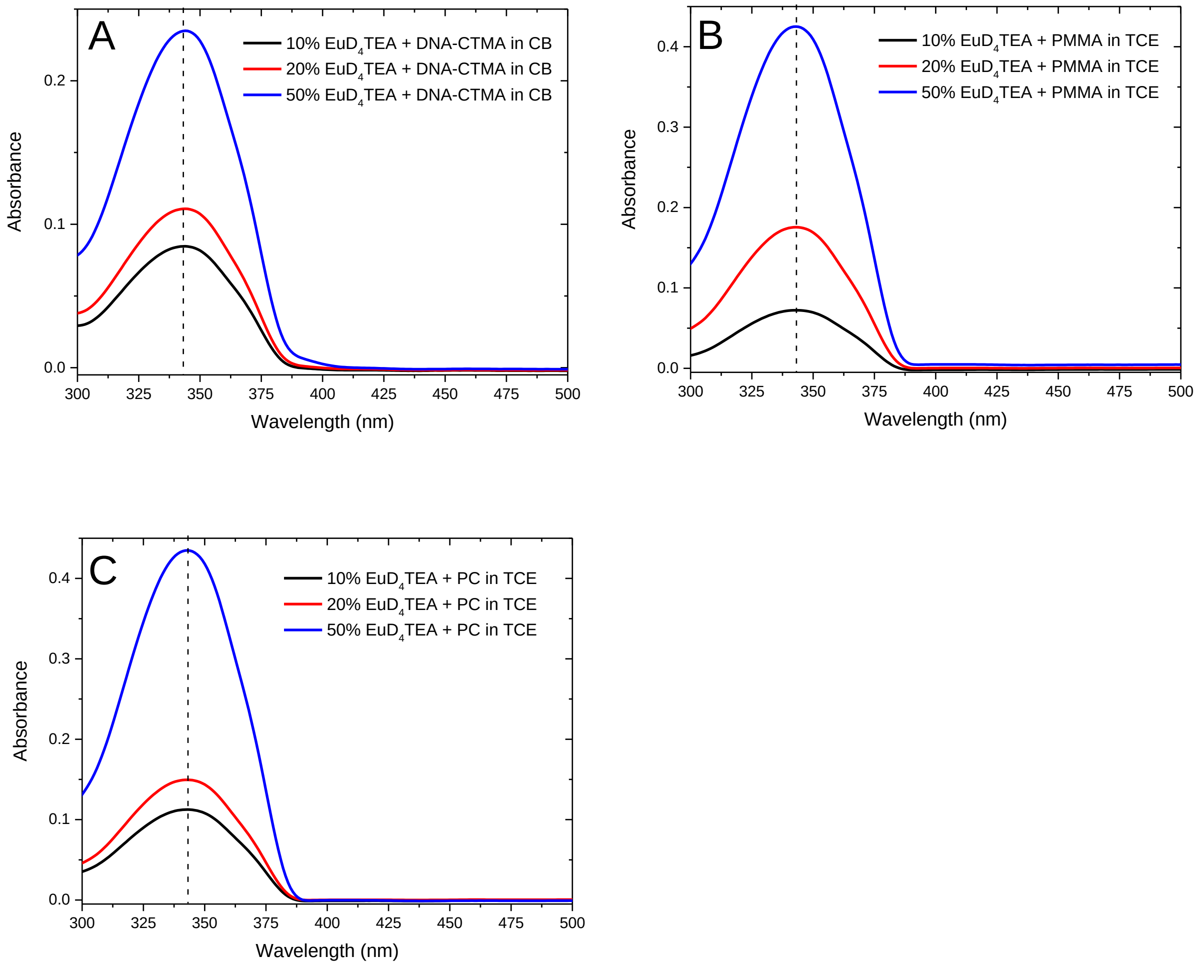


**Figure S1.** Panel A,B and C: solutions of EuD4TEA complex at different concentrations in the TCE and CB solvents in presence of DNA–CTMA, PMMA and PC polymers, respectively.

# III. Photophysical data in thin films

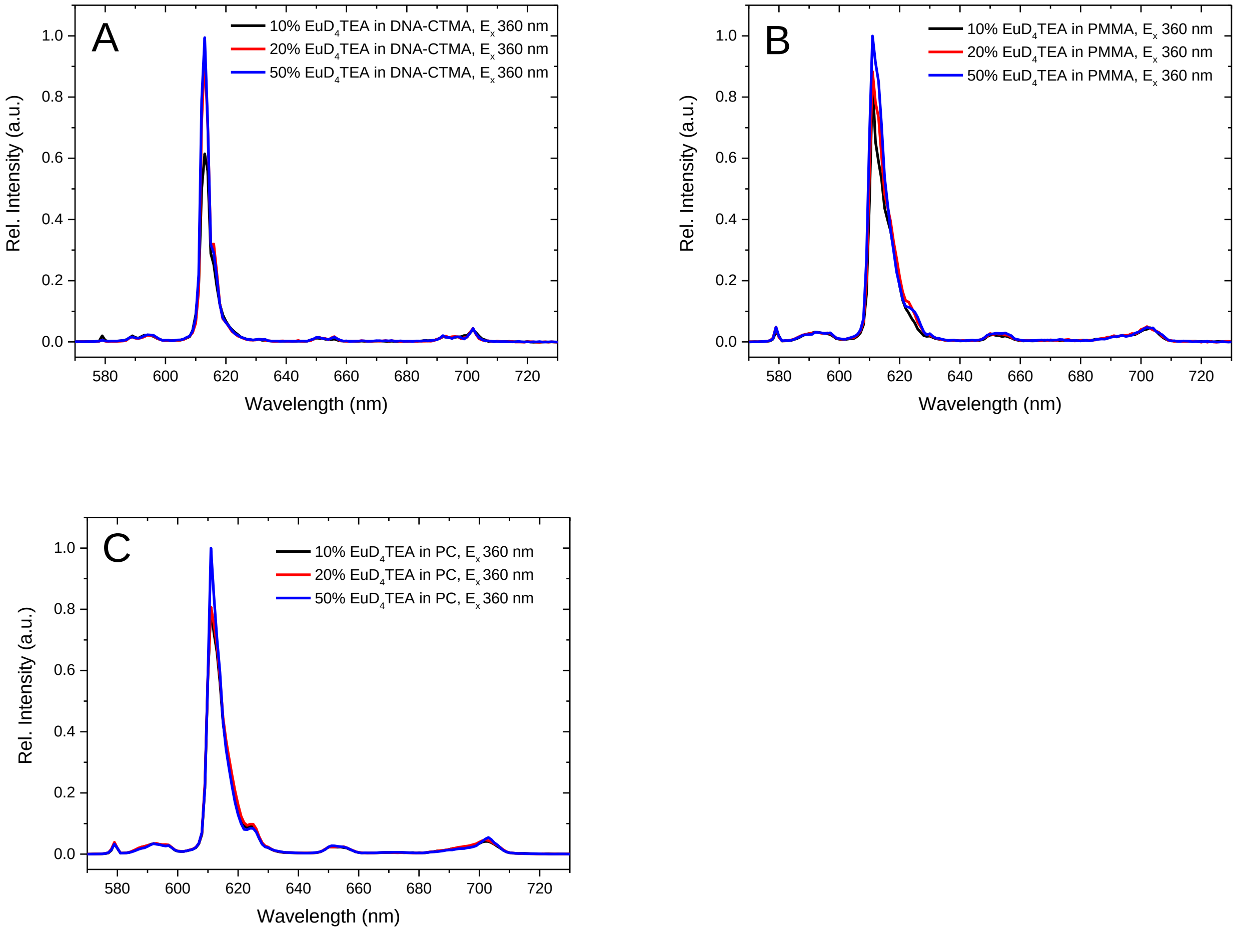


**Figure S2**. Panel A, B, and C emission spectra of thin films of the $EuD_4TEA$ complex at various concentrations in DNA-CTMA, PMMA and PC matrices, respectively. For all three matrixes, the spectra have been scaled such that the respective $^5D_0 \rightarrow {}^7F_1$ bands have identical areas.

Since the $^5D_0 \rightarrow {}^7F_1$ transition is a magnetic dipole, whose intensity is largely independent of the environment of the $Eu^{3+}$ ion, it can be used as an "internal reference". Therefore, for all three matrixes, in order to compere different emission spectra, the latter have been scaled in such a way that the respective $^5D_0 \rightarrow {}^7F_1$ bands have identical areas, see Figure S2.

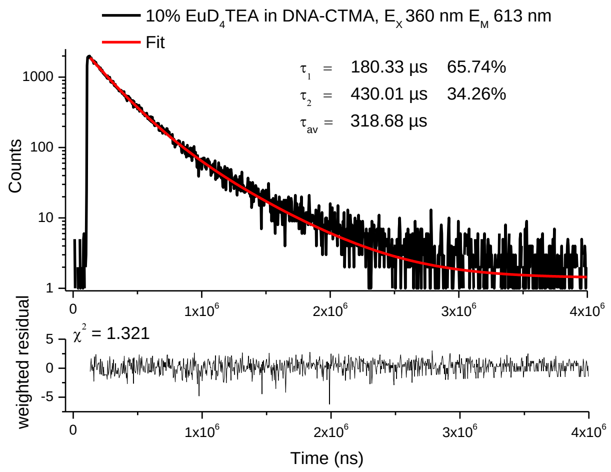


**Figure S3**. Time-resolved fluorescence decays and the relative fit of 10 % EuD4TEA complex in the DNA-CTMA matrix. Emission wavelength 613 nm, excitation wavelength 360 nm.

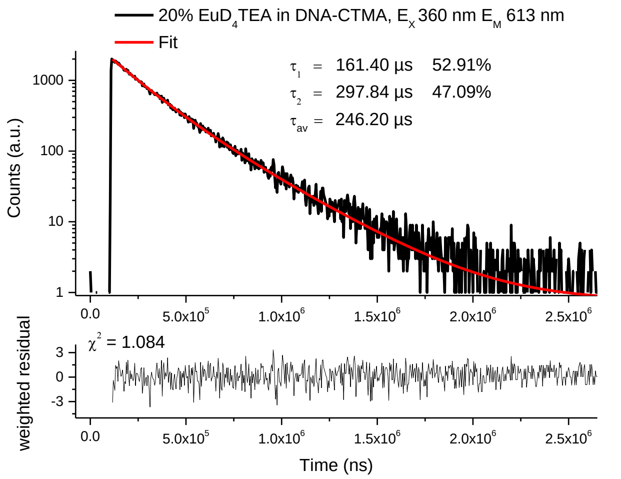


**Figure S4**. Time-resolved fluorescence decays and the relative fit of 20 % EuD4TEA complex in the DNA-CTMA matrix. Emission wavelength 613 nm, excitation wavelength 360 nm.

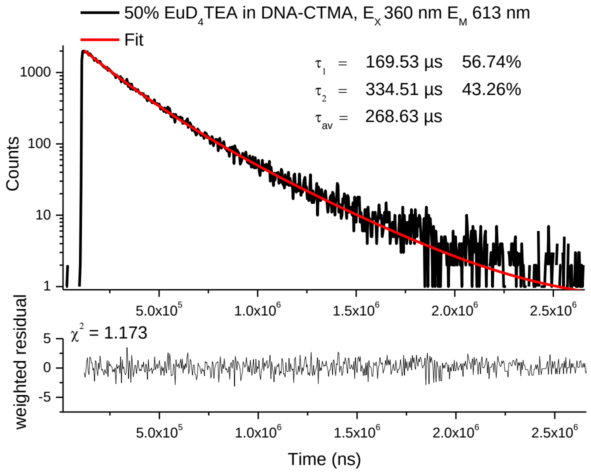


**Figure S5**. Time-resolved fluorescence decays and the relative fit of 50 % EuD4TEA complex in the DNA-CTMA matrix. Emission wavelength 613 nm, excitation wavelength 360 nm.

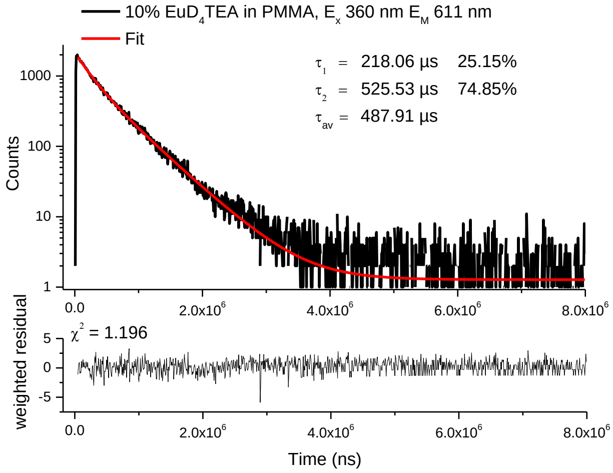


**Figure S6**. Time-resolved fluorescence decays and the relative fit of 10 % EuD4TEA complex in the PMMA matrix. Emission wavelength 611 nm, excitation wavelength 360 nm.

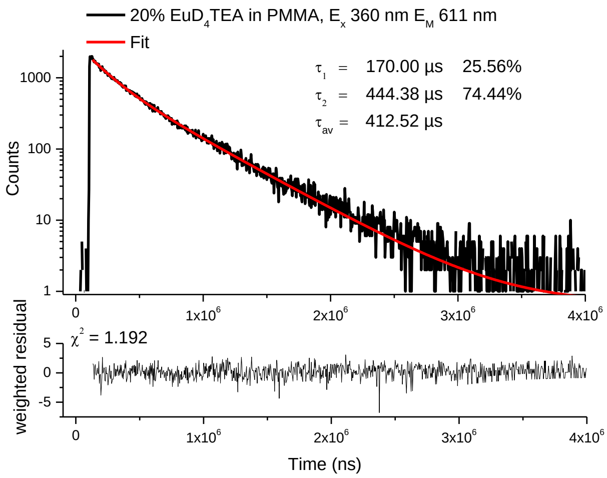


**Figure S7**. Time-resolved fluorescence decays and the relative fit of 20 % $EuD_4TEA$ complex in the PMMA matrix. Emission wavelength 611 nm, excitation wavelength 360 nm.

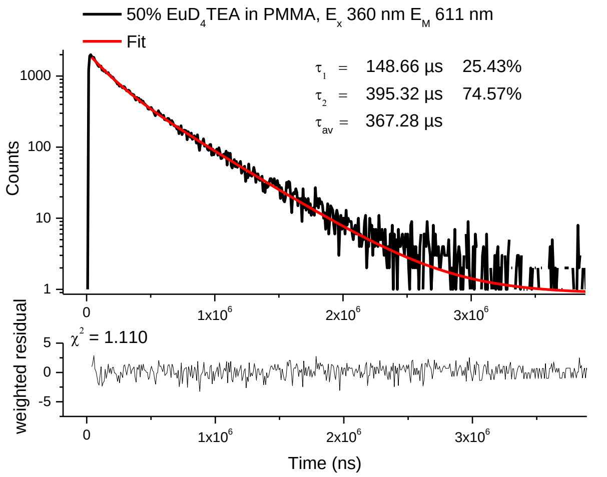


**Figure S8**. Time-resolved fluorescence decays and the relative fit of 50 % $EuD_4TEA$ complex in the PMMA matrix. Emission wavelength 611 nm, excitation wavelength 360 nm.

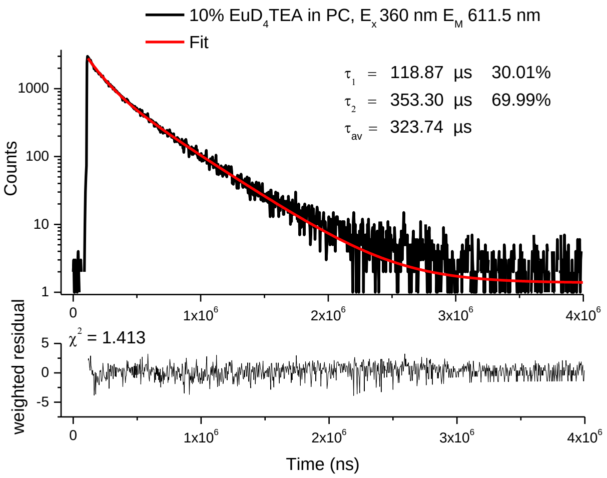


**Figure S9**. Time-resolved fluorescence decays and the relative fit of 10 % $EuD_4TEA$ complex in the PC matrix. Emission wavelength 611.5 nm, excitation wavelength 360 nm.

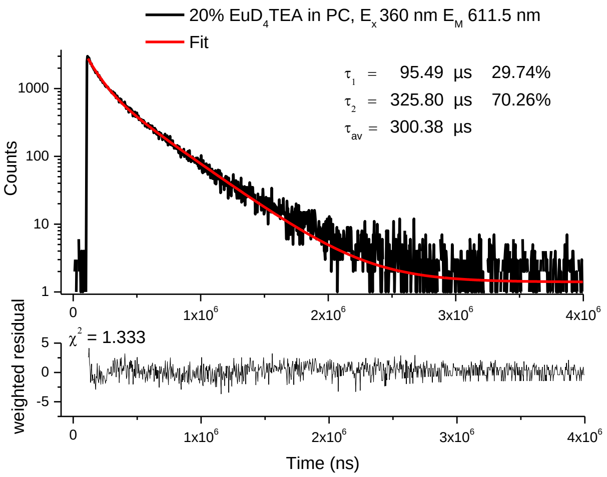


**Figure S10**. Time-resolved fluorescence decays and the relative fit of 20 % $EuD_4TEA$ complex in the PC matrix. Emission wavelength 611.5 nm, excitation wavelength 360 nm.

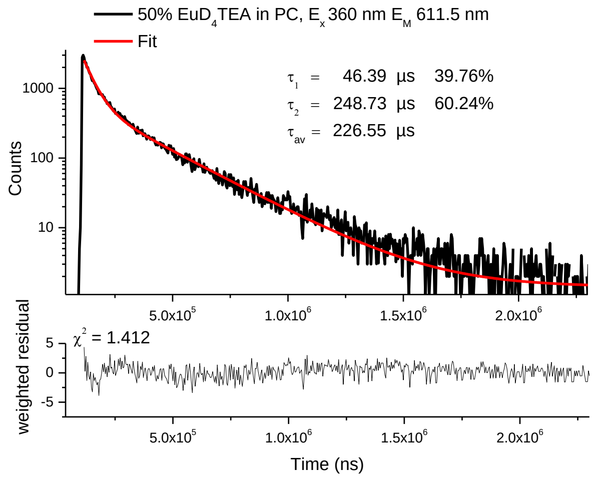


**Figure S11**. Time-resolved fluorescence decays and the relative fit of 50 % $EuD_4TEA$ complex in the PC matrix. Emission wavelength 611.5 nm, excitation wavelength 360 nm.

| ***Sample*** | ***Eu(III):Polymer (weight ratio of Eu(III))*** | ***Lifetime (μs)*** | | ***Amplitude (%)*** | | ***$\tau_{av}$ (μs)*** |
|---|---|---|---|---|---|---|
| | | ***$\tau_1$*** | ***$\tau_2$*** | ***$\alpha_1$*** | ***$\alpha_2$*** | |
| PC/Eu(III) | 1:2 (50 % wt) | 46.39 | 248.73 | 39.76 | 60.24 | 226.55 |
| | 1:5 (20 % wt) | 95.49 | 325.80 | 29.74 | 70.26 | 300.38 |
| | 1:10 (10 % wt) | 118.87 | 353.30 | 30.01 | 69.99 | 323.74 |
| PMMA/Eu(III) | 1:2 (50 % wt) | 148.66 | 395.32 | 25.43 | 74.57 | 367.28 |
| | 1:5 (20 % wt) | 170.00 | 444.38 | 25.56 | 74.44 | 412.52 |
| | 1:10 (10 % wt) | 218.06 | 525.53 | 25.15 | 74.85 | 487.91 |
| DNA-CTMA/Eu(III) | 1:2 (50 % wt) | 169.53 | 334.51 | 56.74 | 43.26 | 268.63 |
| | 1:5 (20 % wt) | 161.40 | 297.84 | 52.91 | 47.09 | 246.20 |
| | 1:10 (10 % wt) | 180.33 | 430.01 | 65.74 | 34.26 | 318.68 |

**Table S1.** Lifetimes and amplitude of the two pre-exponential coefficients ($\alpha_1$ and $\alpha_2$) of the fitting exponential functions of the thin films of the $EuD_4TEA$ complex at various concentrations in DNA-CTMA, PMMA and PC matrixes.

# IV References

46. K. Suzuki, A. Kobayashi, S. Kaneko, K. Takehira, T. Yoshihara, H. Ishida, Y. Shiina, S. Oishic, S. Tobita, Reevaluation of absolute luminescence quantum yields of standard solutions using a spectrometer with an integrating sphere and a back-thinned CCD detector, Phys. Chem. Chem. Phys., 2009, 11, 9850–9860. DOI: 10.1039/b912178a
